# Direct mapping of electrical noise sources in molecular wire-based devices

**DOI:** 10.1038/srep43411

**Published:** 2017-02-24

**Authors:** Duckhyung Cho, Hyungwoo Lee, Shashank Shekhar, Myungjae Yang, Jae Yeol Park, Seunghun Hong

**Affiliations:** 1 Department of Physics and Astronomy, and Institute of Applied Physics, Seoul National University, Seoul 151-747, Korea; 2 Department of Automotive Engineering, Doowon Technical University College, Anseong 456-718, Korea; 3 Department of Biophysics and Chemical Biology, Seoul National University, Seoul 151-747, Korea

## Abstract

We report a noise mapping strategy for the reliable identification and analysis of noise sources in molecular wire junctions. Here, different molecular wires were patterned on a gold substrate, and the current-noise map on the pattern was measured and analyzed, enabling the quantitative study of noise sources in the patterned molecular wires. The frequency spectra of the noise from the molecular wire junctions exhibited characteristic 1/*f*^2^ behavior, which was used to identify the electrical signals from molecular wires. This method was applied to analyze the molecular junctions comprising various thiol molecules on a gold substrate, revealing that the noise in the junctions mainly came from the fluctuation of the thiol bonds. Furthermore, we quantitatively compared the frequencies of such bond fluctuations in different molecular wire junctions and identified molecular wires with lower electrical noise, which can provide critical information for designing low-noise molecular electronic devices. Our method provides valuable insights regarding noise phenomena in molecular wires and can be a powerful tool for the development of molecular electronic devices.

Molecular wires (MWs) are the basic building blocks of molecular electronic devices^1^,^2^,^3^,^4^,^5^,^6^. Understanding the charge transport phenomena across metal-MW-metal junctions is a crucial step for the realization of novel electronic devices based on a single or few MWs^1^,^2^,^3^,^4^,^5^,^6^,^7^,^8^,^9^,^10^,^11^. Extensive efforts have been devoted to the study of MW junctions, revealing their various characteristic properties^1^,^2^,^3^. In particular, noise phenomena, such as the random fluctuations in the electrical currents in MW junctions, have attracted increasing interest as the measurement and analysis of the noise were found to provide useful clues for understanding various aspects of MW junctions^12^,^13^,^14^. In previous noise studies on MW junctions, one had to fabricate and measure multiple MW junctions based on different MWs to compare the noise characteristics of the different MWs. However, it is difficult to fabricate multiple MW junctions with a uniform quality and even MW junctions based on identical MWs often exhibit variations of several orders of magnitude in their current and noise levels^15^,^16^. Due to such problems, it has been extremely difficult to reliably identify the effect of MWs on the current and noise generated in MW junctions, which has been one of the major hurdles for studying the electrical transports by MWs^15^,^16^.

Herein, we report a noise mapping strategy to reliably identify and map the sources of electrical noises in various MW junctions. In this strategy, we patterned different MW junctions in a single metal substrate via a microcontact printing method and mapped the electrical currents and noise power spectra of the sample. Here, the different MW junctions were based on the same substrate and mapped via a single scanning probe, minimizing the possible variations from the substrate and probe and enabling reliable studies on the effect by MWs in the junctions. Analysis of the mapping data indicated that the MW junctions exhibit a characteristic 1/*f*^2^ noise behavior regardless of MW species, unlike metal-metal nanojunctions showing common 1/*f* noise. This behavior was used to identify electrical signals originating from the MWs. Furthermore, the method was used to study the MW junctions of various thiol molecules on a gold substrate. The results indicate that the electrical noise in the junctions mainly originated from the fluctuations of thiol bonds between the MWs and the gold surface. In addition, we quantitatively compared the bond-fluctuation frequencies of different MWs and identified the MWs with lower noise. These results show that the electrical fluctuations measured by our method can provide useful insights regarding the physical properties of MW layers and clues to understand the behavior of individual MWs. Considering that the electrical noise has been one of the most difficult problems to study and is critical for molecular electronic devices, our results are expected to be a major breakthrough in molecular electronics research and may have a significant impact on the development of molecular electronic devices.

## Results and Discussion

### Experimental setup

**Figure 1:**
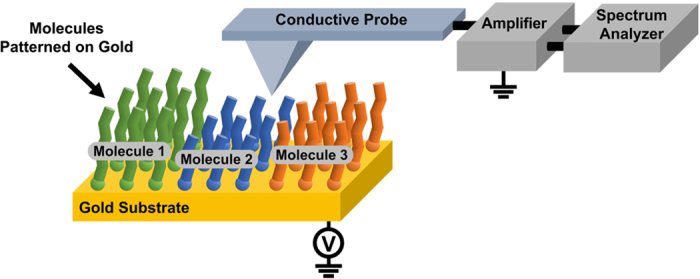
Schematic diagram depicting the experimental setup for measuring the electrical current and noise in patterned layers of molecular wires. A Pt-based conducting probe installed on an AFM was placed on the patterns of molecular wire layers, and a bias voltage was applied between the probe and underlying gold substrate. The contact force between the probe and molecular wires was maintained at 1 μN using a force feedback loop in the AFM system. The electrical currents and noise PSDs of the currents were simultaneously measured using a preamplifier and homemade spectrum analyzer. The maps of the electrical currents and noise PSDs were obtained by scanning the probe above the patterned surface.

Figure 1 shows a schematic diagram of our experimental setup for the noise and current measurements on a patterned layer of MWs. The details of the experimental procedures are described in the methods section. In brief, we prepared the self-assembled monolayer (SAM) patterns of different MWs on a gold substrate via a microcontact printing method^17^. Then, a Pt-based conducting probe installed on a contact mode atomic force microscopy (AFM) system approached the sample, forming a metal (gold)/MW/metal (Pt probe) junction. Here, the contact force between the probe and MW layer was maintained at 1 μN using the contact force feedback loop of the AFM system. To map the electrical current and noise, a bias voltage was applied between the probe and gold substrate with force feedback, and the currents through the gold/MW/Pt probe junction were measured. The measured currents were converted to amplified voltage signals by a preamplifier (SR570, Stanford Research Systems). Subsequently, the noise PSD of the signals in a specific frequency range was measured using a homemade spectrum analyzer. Two-dimensional maps of the currents and noise PSDs could be obtained by scanning the probe on the MW patterns during the measurement. The typical scan speed was 0.5 μm/s. Then, the measured *current* and *PSD* maps were analyzed to obtain maps of *the resistances* and *the mean-square fluctuations of resistances* of the patterned MWs, respectively. Note that the characteristics of the scanning probe-based MW junctions could vary due to the variations of the probes and substrates used. However, with our strategy, we could measure the noise characteristics of different MWs using a single probe on the same substrate. Thus, the possible variations of the MW junctions, which have often been a serious problem in previous works, were minimized, enabling reliable comparative studies of the different MWs.

### Characteristic scaling behavior of current noise in molecular wire-metal probe junctions

**Figure 2:**
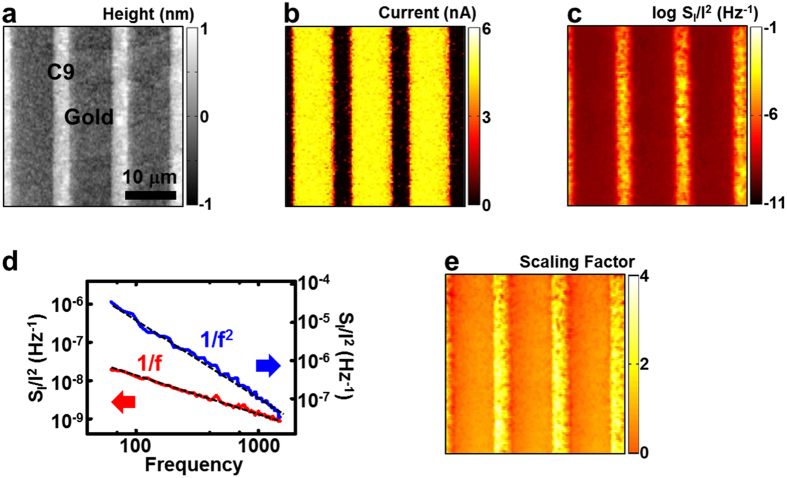
Characteristic scaling behavior of current noise in molecular wire-metal probe junctions. (**a**) AFM topography image showing the line-shaped patterns of 1-nonanethiol (C9) SAMs on a gold substrate. (**b**) Current map of the SAM patterns. (**c**) Current-normalized noise PSD map of the SAM patterns. (**d**) Graph showing noise PSD versus frequency data measured in a gold (red) and a molecular wire layer (blue) region on the sample. The data were fit to a *1/f*^*β*^ curve with different values of the scaling factor *β*. The noise PSD spectra from a molecular wire region were fit to a *1/f*^*2*^ curve corresponding to the scaling factor of approximately 2.0, whereas those from the gold region exhibited a typical *1/f* behavior with a scaling factor of approximately 1.0. (**e**) Scaling factor map of the noise PSD spectra measured on the SAM patterns. The *molecular wire* and *gold* regions exhibited scaling factors of *~2.0* and *1.0*, respectively.

Figure 2a and b show the AFM topography and the electrical current maps measured on 1-nonanethiol (C9) SAM patterns on a gold substrate. A bias voltage of 5 mV was applied between the gold substrate and conducting AFM probe to measure the current map. The widths of the *C9* and *gold* regions were ~*4* and *8* μm, respectively. The measured height of the C9 monolayer was approximately 1.1 nm, which is consistent with the reported length of a C9 molecule^18^. The topography and current images showed no bumps or cracks, indicating the high quality of the SAM of the C9 molecules. Any mechanical or chemical distortions on the probe during scanning could result in abrupt changes or distortions in the images. In such cases, the data were discarded, and the probe was replaced with a clean one. We could repeatedly image a specific region of a molecular pattern more than 5 times without abrupt changes or distortions in the imaging results. This indicates the distortion on the probe or samples during the scanning was minimal, and at least in the presented image, we performed the noise measurement while maintaining the same probe conditions. The electrical current level in the MW regions was approximately 100 times lower than that in the Au regions, implying that the voltage drop mainly occurred at the MW junctions in our measurement system. In addition, we could measure a stable current level for a relatively long time period when the probe was placed at a fixed position with the AFM force feedback on (Fig. S1 in the Supplementary Information). Such clear current images and stable current levels indicate electrically clean MW layers and stable electrical contacts during our measurements.

We mapped the noise PSD values (*S*_*I*_) at a specific frequency while measuring the topography and current maps via noise microscopy. We obtained a current-normalized noise PSD (*S*_*I*_/*I*^2^) map by dividing the *S*_*I*_ values in the *S*_*I*_ map by the corresponding current (*I*) values in the current map. The *S*_*I*_/*I*^2^ value is a useful parameter to represent the noise level of an electrical channel^19^. Figure 2c shows the *S*_*I*_/*I*^2^ map at 31.6 Hz. The noise level of the MW (~10^−5^ Hz^−1^) was considerably higher than that in the bare gold region (~10^−9^ Hz^−1^). The low noise level in the gold region indicated negligible background noise in our noise microscopy system. In contrast, the high noise level in the C9 monolayer region implies that the C9 MWs between the gold and AFM probe were generating a significant amount of electrical noise. As a control experiment to check the reliability of our electronics, we used our electronics (without the AFM system) to measure the electrical noise from a dummy resistor (1.0 GΩ glass glaze film resistor) whose resistance was similar to that of the C9 MW junction (Fig. S2 in the Supplementary Information). The noise level of the dummy resistor was ~10^−7^ Hz^−1^, which was 100 times smaller than that from the C9 junction. This result implies that the MWs generated a significant amount of electrical noise, even compared with other high-resistance materials, and our system can be used to reliably measure such noise from MW junctions.

In our setup, we placed a conducting probe at a fixed location on the MW-patterned sample and measured the noise spectra from that location. Figure 2d shows the current-normalized noise PSD spectra that were measured at two different regions: (i) a bare gold region (red line) and (ii) a C9 SAM region (blue line). In both cases, the noise PSD values were quite high at low-frequency conditions and decreased with increasing frequency following the power laws, indicating the existence of frequency-dependent noise sources other than the frequency-independent Johnson–Nyquist noise sources in the sample. We fitted the noise PSD spectra using functions of frequency *f* in the form of *PSD = A/f*^*β*^, where *A* and *β* are the fitting parameters. The fitting curves are marked by black dashed lines in the Fig. 2d. Here, the fitting parameter *β* gives useful information about how fast the noise PSD decreased with the increasing frequency, and it was named as the “scaling factor”. As indicated by the fitting curve (dashed line), the PSD measured at the gold region showed a typical *1/f* noise behavior with *β* ~1. In contrast, a 1/*f*^*2*^ noise behavior with *β* ~2 was observed when we measured noise spectra in the C9 region.

Previous works have shown that if a noise source can generate a two-state conductance fluctuation between a conducting state (*C*) and non-conducting state (*N*), the electrical noise generated by the noise source should have a Lorentzian form like^15^,^20^,^21^,


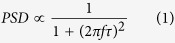


Here, *τ* is the characteristic time of the fluctuation and is defined as (*λ* + *μ*)^−1^, where *λ* and *μ* are the transition rates of the *C-to-N* and *N-to-C* transitions, respectively. At a rather high-frequency condition of *2πfτ* ≫ 1, the PSD can be approximated to be proportional to *1/f*^*2*^^13^,^15^. Thus, 1/*f*^2^ noise in the C9 monolayers could be generated by the uniform Lorentzian noise sources in the MW layer^13^. The measured PSD on the C9 monolayer was fitted to the Lorentzian curve, allowing us to estimate the time constant *τ* as 11 ms (Fig. S3 in the Supplementary Information), which is consistent with a previously reported value^13^. In contrast, if a current channel had a large number of noise sources with different characteristic times, the PSDs of the different noise sources were summed together, resulting in the total noise generated with a different scaling factor^15^. For example, consider a conducting channel with a large number of noise sources generating Lorentzian noise. If the distribution function of *τ* is proportional to 1/*τ*, the total noise PSD of the channel is proportional to 1/*f*^15^,^22^. Actually, 1/*f* noise has been commonly observed in various metallic and semiconducting channels, and different possible noise sources have been suggested to interpret such noise^22^,^23^,^24^. For example, an explanation for the 1/*f* noise in metallic films, such as the gold film in our work, is the scattering of electrons by mobile defects^22^. This characteristic feature of the scaling factors of the noise from MW junctions could be used to qualitatively distinguish the noise of MW junctions from that of other metallic junctions.

We measured the scaling factor *β* at each point on the C9 patterns to obtain a scaling factor map (Fig. 2e). Here, the noise PSD was measured in the frequency range of 5.47–173 Hz at each point of the surface and used to estimate the scaling factors. The scaling factor map shows a clear contrast between the molecular region (*β*~2.0) and gold region (*β*~1.0). We obtained consistent results from all different MW samples presented in this manuscript, indicating that the scaling analysis of the noise spectra is a simple yet powerful means to identify the noise generated by MWs. Considering that it is often difficult to distinguish the noise generated by MWs from that generated by metal electrodes in MW junctions, this result can provide an important tool for the research concerning charge transport phenomena in MW junctions.

### Mapping of mean-square fluctuations of the molecular resistances on SAM patterns comprising multiple molecular species

**Figure 3:**
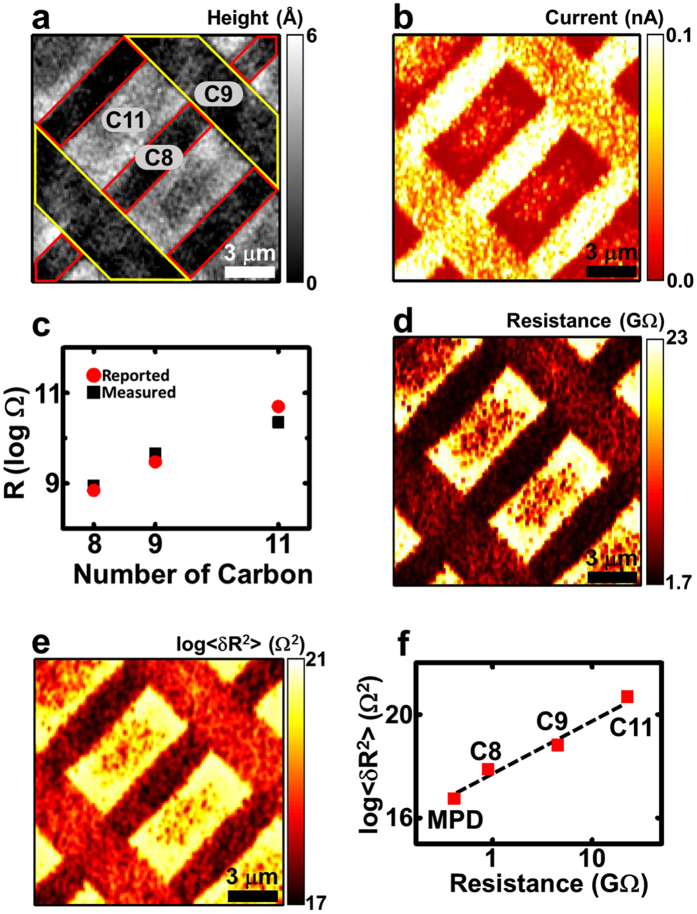
Mapping of the mean-square fluctuations of molecular resistances <*δR*^*2*^> on SAM patterns comprising three different molecular wires with different lengths on a gold substrate: 1-octanethiol (C8), 1-nonanethiol (C9), and 1-undecanethiol (C11). (**a**) AFM topography image. The areas surrounded by the *red* and *yellow* lines were coated with the SAMs of *C8* and *C9* molecular wires, respectively. The other regions were covered with C11 SAMs. (**b**) Current map. A bias voltage of 5 mV was applied between the probe and gold substrate. (**c**) Reported (red circles) and measured (black squares) resistance values of individual C8, C9, C11 molecules. (**d**) Map showing the resistance of individual molecular wires in the SAM patterns. (**e**) Map showing the mean-square fluctuations of molecular resistances, *<δR*^*2*^*>*, obtained from the PSD and current images. Importantly, we could quantitatively compare the *< δR*^*2*^*>* values of different molecular wires using the map. (**f**) Graph of *<δR*^*2*^*>* versus the molecular resistance. The *<δR*^*2*^*>* was proportional to the squared molecular resistance *R*^*2*^.

Figure 3a and b show the topography and current images of a molecular pattern comprising three different thiol molecular species with different chain lengths: 1-octanethiol, 1-nonanethiol, and 1-undecanethiol (C8, C9, and C11). The detailed patterning processes are presented in the methods section. Briefly, C9 SAMs were first patterned on a gold substrate via the microcontact printing method (the regions in yellow trapezoids). Then, C8 SAMs (the regions surrounded by red lines) were patterned in a direction perpendicular to the C9 SAMs. Finally, the patterned substrate was dipped in a 0.2 mM solution of C11 molecules such that the C11 molecules filled up the remaining bare gold region. The topography image shows that the C11 SAMs were higher than the other SAMs about ~0.4 nm, and the average height difference between the C8 and C9 SAMs was approximately 0.1 nm, which is consistent with previously reported results^16^. The current map shows clear differences in the current levels between the C8, C9 and C11 regions. An MW with a longer chain length exhibited a lower current level than an MW with a shorter chain length. Previous reports showed that tunnel-currents through MW SAMs decrease exponentially with increasing molecular lengths, and thus, the current levels were extremely sensitive to the molecular lengths, which is also consistent with our results^16^,^25^,^26^.

When a conducting probe with a contact area *A* and a known bias voltage (*V*) was used to measure the electrical currents (*I*) through a layer of MWs, the measured junction resistance (*R*_junction_) of the substrate-MWs-probe junction can be written using the resistance of a single MW (*R*_MW_) as follows (considering parallel MWs between two electrodes):


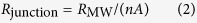


where *n* represents the number of MWs in the unit area of the MW layer. Inversely, the *R*_MW_ could be obtained from the *R*_junction_ (=*V*/*I*) as follows:


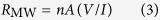


In the case of alkanethiol monolayers, the *n* value has been reported as ~4.65 molecules/nm^2^ 27. Here, the contact area *A* of the conducting probe is still an unknown parameter. However, because we used the same probe to measure the electrical currents from different MWs (Fig. 3b), the values of *A* for the measurements on different MWs were identical, and we could estimate the value of *A* by using it as a fitting parameter. The red circular dots in Fig. 3c indicate previously reported values of single MW resistance from C8, C9, and C11 MWs^28^. Using Eq. (3) with *A* as a single fitting parameter, the reported resistance values fit our measured data (black square dots in Fig. 3c) extremely well. The contact area *A* estimated from the fitting procedure was ~9.6 nm^2^, which is also consistent with the dimension of our conducting probe^29^.

Using the current map in Fig. 3b and the estimated contact area *A*, we obtained the map of the single-molecule resistance values (*R*_individual_) in the different molecular layers (Fig. 3d). In the remainder of this paper, we indicate the resistance of the individual MWs (*R*_MW_) as *R* for simplicity. The single-molecule resistance values of *C8, C9* and *C11* MWs were ~*0.9, 4.5* and *22.5* GΩ, respectively. The scaling factor *β* of the noise-frequency spectra measured on these different MW regions exhibited a value of approximately 2.0, indicating that we were measuring the currents and resistances originating from the MWs rather than metal-metal nanocontacts. In our experiment, because a single probe was used to measure the resistance values of different MWs under the same conditions, the possible variation of the MW junctions was minimized, and we can precisely estimate the ratio of the molecular resistance values of the different MWs in Fig. 3d.

The electrical noise generated in the MW junctions is an important parameter that should determine the performance of MW-based devices. In addition to the electrical currents, we also measured the map of the electrical current noise in the layers of different MWs (Fig. S4 in the Supplementary Information). Furthermore, we propose a theoretical model to quantitatively estimate the mean-square fluctuations of the single-molecule resistances of the MWs. Previous studies suggested that an MW junction can exhibit a Lorentzian resistance fluctuation^15^,^25^. For example, the sudden break and reconnection of a bridge molecule in an MW junction can induce a two-state conductance fluctuation between a conducting and non-conducting state, resulting in the Lorentzian fluctuation of the junction resistance with a characteristic time *τ*. At a rather high frequency *f*, the PSD (*S*_*R*_) of such resistance fluctuations in the MW junction can be written as^30^


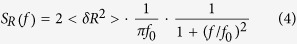


Here, *<δR*^*2*^*>* and *f*_*0*_ are the mean-square fluctuations of *the molecular resistance* and 1/(2*πτ*), respectively. Previous studies have shown that electrons tunneling through an MW junction can provide energy to the junction by interacting with phonons^21^. Further, it has been suggested that the energy from the tunneling carriers is the main source of the energy required for the resistance fluctuation of an MW junction, and thus, the rate of the fluctuations should be proportional to the amount of current through the junction^21^,^30^. Thus, with a constant *B, f*_*0*_ can be written as^30^


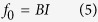


Here, *I* denotes the current through an MW. Using Eq. (5) and the measured values of *f*_*0*_ and *I*, we calculated the constant *B* as ~1.3 × 10^4^ Hz/nA. At high-frequency conditions of *f/f*_*0*_ >>1, Eq. (4) can be rewritten as


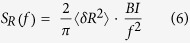


The noise PSD values of the fluctuations of the resistance and current through an MW are related by^20^


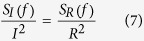


and from Eq. (6), *<δR*^*2*^*>* can be written as


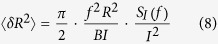


Figure 3e shows the map of *<δR*^*2*^> obtained after using Eq. (8). The *S*_*I*_*/I*^*2*^ map in Fig. S4 was used for the calculation. Because the measured *S*_*I*_ and *I* values should be proportional to the number of MWs (*nA*) between the probe and gold^20^, the measured *S*_*I*_/*I*^2^ values should be proportional to 1/(*nA*). Thus, we multiplied the values in the measured *S*_*I*_/*I*^2^ map by *nA* to obtain *S*_*I*_/*I*^2^ values for an ‘individual’ MW. In addition, the current through a single MW was obtained by dividing the current values in the measured current map by *nA*. The *<δR*^*2*^*>* map shows the clear differences between the *<δR*^*2*^*>* levels for different MWs. The <*δR*^*2*^ > values in the *C8, C9* and *C11* regions were ~*7.6* × *10*^*17*^, *6.7* ×* 10*^*18*^ and *4.8* × *10*^*20*^ Ω^2^, respectively. We are the first to have quantitatively measured and compared the mean-square fluctuations of the molecular resistances of different MWs, which should be a significant breakthrough for noise studies on MWs.

Figure 3f shows the *<δR*^*2*^*>* values as a function of the molecular resistance *R*. Here, the *<δR*^*2*^*>* value of the 4-mercaptopyridine (MPD) MW was estimated from the *S*_*I*_*/I*^*2*^ map measured on an MPD/C9 patterned sample (Fig. S5 in the Supplementary Information). As indicated by the dashed line, <*δR*^*2*^> was found to be proportional to the squared molecular resistance (*R*^2^) as


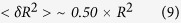


Although the MPD and other alkanethiol MWs had completely different molecular chains with different lengths and structures, they still exhibited a similar behavior. Previous works have shown that the resistance of an insulating MW with length *d* can be written as^31^


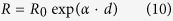


Here, *R*_*0*_ corresponds to the contact resistance, which should depend on the metal-molecule bonds and the contact between the molecules and AFM probe^31^. Because we used the same probe and molecules with the same thiol bonds, the contact resistance *R*_*0*_ should be highly similar for all MWs in the graph^31^. The variable *α* is the tunneling attenuation factor, which depends only on the chain of the MWs^31^. From Eq. (10), the mean-square fluctuation *<δR*^*2*^*>* of the molecular resistance values, which is responsible for the current noise in the MW junctions, can be written as


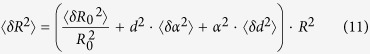


Here, the first term originates from the molecular contact resistance and its fluctuations^32^,^33^. In our case, because we used the same AFM probe and MWs with the same thiol bonds, the first term should be highly similar for all molecular species in Fig. 3f. The second and third terms originate from the properties of the molecular chains and their fluctuations, such as molecular length fluctuations and chain twisting^34^. Because the conjugated MPD and other alkanethiol MWs in our experiment had different molecular chains, these terms should vary significantly depending on the molecular species. However, our results indicate that 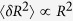 even though our molecular species had quite different chains. This indicates that the electrical noise of the MW junctions in our experiments mainly originated from the fluctuation of the metal-molecule bonds. Although extensive efforts have been made to understand the charge transport and noise in MW junctions, the origin of the dominant noise source in the junctions remains uncertain^21^. Our results imply that the electrical noise in MW junctions mainly originates from the fluctuation of molecular bonds, which is a valuable insight into the transport phenomena of MW-based devices.

### Mapping the spectral noise characteristics of SAM patterns comprising molecular wires with different chain structures

**Figure 4:**
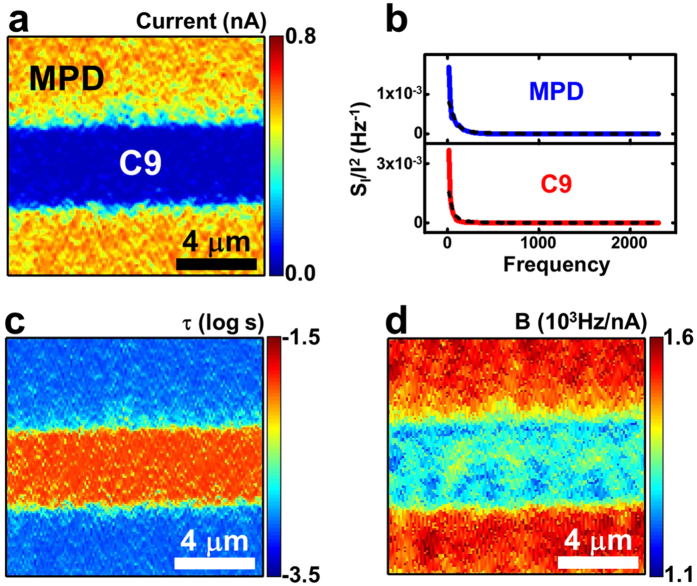
Noise spectral characteristics of MPD and C9 molecules. (**a**) Current map measured on the patterns of the MPD (yellow region) and C9 (blue region) molecules. (**b**) Current-normalized PSD spectra of the MPD (blue lines) and C9 (red lines) molecules and their Lorentzian fittings (black dashed lines). (**c**) Map of *τ* measured on the patterns of the MPD (blue region) and C9 (orange regions) molecules. The MPD regions exhibited shorter *τ* values (~0.9 ms) than the C9 regions (~12 ms), indicating more frequent bond fluctuations on the MPD molecular junctions. (**d**) Map of the proportional factor *B* measured on the patterns of the MPD (red or yellow regions) and C9 (blue or emerald region) molecules. The MPD region exhibited higher *B* values than the C9 region, implying that the bond-fluctuation events with the same currents occur more frequently in the MPD molecules than in the C9 molecules.

The spectral analysis of the noise spectrum was used to reveal the more detailed characteristics of the MW-based junctions (Fig. 4). Figure 4a shows the current map obtained from the patterns of the conjugated MPDs and alkane chain-based C9 molecules. Because the MPD molecule has conjugated backbones with herringbone structures and a shorter chain length than the C9 molecule, the current level of the MPD regions appeared to be considerably higher than that on the C9 regions. Figure 4b shows the current-normalized PSD (*S*_*I*_*/I*^*2*^) spectra that were measured in an MPD region (blue line) and a C9 region (red line). Both regions exhibited 1/*f*^2^ noise characteristics. The PSD spectra on the *MPD* and *C9* regions were fitted well using Lorentzian curves (black dashed lines) with fitting parameters *τ* of ~*1.3* ms and *11.5* ms, respectively. The results are compatible with previously reported values^13^,^30^. Here, the fitting parameter *τ* is the characteristic time, which should be proportional to the mean time interval between each bond-fluctuation event. Thus, the shorter *τ* of the MPD molecules indicated that the bond-fluctuation events occurred more frequently in the MPD molecules than the C9 molecules. One plausible explanation is the larger currents in the MPD molecular regions because the energy needed for the bond fluctuations is largely provided by the current passing through the MW junctions^21^,^30^. Furthermore, the relatively low packing density and rather high defect densities of the MPD layers may have contributed to more frequent bond fluctuations^21^.

For a more detailed analysis, we obtained the map of *τ* on the molecular layers (Fig. 4c). By inserting Eq. (5) and the fitting results from Eq. (9) into Eq. (8)*τ* can be written as


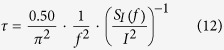


Utilizing above equation, we could calculate *τ* on each point of the MPD/C9 patterns to obtain a *τ* map (Fig. 4c) from the PSD and current maps. The averaged *τ* values on the *MPD* and *C9* regions were ~*0.9* and *12* ms, respectively. Although the fitting processes were quite different, the *τ* values estimated from the *S*_*I*_/*I*^2^ values in the noise mapping data were comparable to the *τ* values estimated from the fitting of the noise spectra in Fig. 4b, indicating the reliability of our analysis method. The map shows that the molecular bonds in the MPD layers fluctuate more frequently than those in the C9 layers. The map of *τ* can also be used to distinguish some alkanethiol molecules. The *τ* map for the patterns of C8, C9, and C11 (Fig. S6) shows that the C8 and C9 layers had more frequent bond fluctuations than the C11 layer. Because alkanethiol molecules with longer alkane chains have larger chain-chain interactions than alkanethiol molecules with shorter chains, they likely formed well-ordered lattice structures on the solid substrates and exhibited a reduced frequency of bond fluctuations^35^. These results show that the *τ* mapping can be a useful tool to discriminate between the different molecular species and their stability on solid substrates.

Because the fluctuations in the MW junctions were mainly triggered by the currents, as described in Eq. (5)^21^,^30^, *τ* can be written as


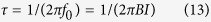


Here, the proportionality factor *B* represents the frequency of the molecular bond fluctuations when a specific amount of electrical current passes through, and thus, it can be a useful characteristic parameter for demonstrating the noise properties of specific MWs. We estimated the map of *B* on the MPD/C9 patterns using the *τ* map, the current map and Eq. (13) (Fig. 4d). The average values of *B* in the *MPD* and *C9* regions were ~*1.52* × *10*^*3*^ and *1.29* × *10*^*3*^ Hz/nA, respectively. These results indicate that with the same amount of current, the molecular bonds of the MPD molecules fluctuate more frequently than those of the *C9* molecules. A previous study suggested that the characteristic frequency of the noise in molecular layers will depend on how densely the molecules cover the gold substrate^21^. For example, in case of a densely packed molecular layer, the lateral motion of the Au–S bonds was hindered, and the number of bond-fluctuation events per unit-time was reduced compared to a loosely packed molecular layer^21^,^36^. It was also reported that the packing density of the MPD molecule with a pyridine ring is lower than that of the C9 molecule^37^. Thus, the higher *B* value of the *MPD* molecule in our data could be attributed to the lower packing density of the MPD molecules than that of the *C9* molecules. Our results illustrate that we can expect more molecular bond fluctuations and thus electrical noise with a higher frequency in the MPD-based electrical junctions than in the C9-based electrical junctions. The noise characteristics of the MWs directly affect the quality of the MW junctions and eventually determine the performance of the MW-based devices. Our method allowed us to quantitatively compare the characteristic parameters determining the electrical noise in MW junctions and even identify the MW species with lower noise under the same conditions. These results should provide an important guideline for developing low-noise molecular electronic devices.

In summary, we have developed a noise mapping strategy for the reliable identification and analysis of electrical noise in MW junctions. In this strategy, we patterned different MWs on the same gold substrate and placed a conductive AFM probe on the pattern to measure the electrical currents and noise PSD spectra simultaneously. By scanning the probe above the MW patterns during the measurement, maps of the *electrical currents* and *noise PSDs* were obtained and used for the comparative analysis of the *electrical resistance values* and the *noise characteristic parameters* in different MWs, respectively. Importantly, we observed a characteristic 1/*f*^2^ noise behavior in the noise PSDs measured from the MW junction, whereas a simple metallic junction exhibited a typical 1/*f* noise behavior. This characteristic behavior was used to qualitatively distinguish the electrical signals of the MW junctions from those of metallic junctions. In addition, we successfully mapped the mean-square fluctuations <*δR*^2^> of the molecular resistances in thiol MW junctions and found that <*δR*^2^> was proportional to the squared molecular resistance *R*^2^, indicating that the noise in the junctions originated mainly from the fluctuation of the thiol bonds on the Au electrode surface. Furthermore, we quantitatively compared the frequency of such bond-fluctuation events and identified MWs with lower electrical noise, which could be an important guideline for designing low-noise molecular electronic devices. Considering that it has been extremely difficult and time consuming to measure the noise characteristics of MWs reliably in the past, our strategy can be a powerful tool and should be a significant breakthrough for the basic research and device applications of MW-based devices.

## Methods

### Preparation of a self-assembled monolayer based on molecular wires

As a substrate, gold films were prepared on SiO_2_ substrates using a thermal evaporation method. Typically, 10 nm of Ti was first deposited as an adhesion layer with a background pressure of approximately 3 × 10^−6^ Torr and a deposition rate of ~1 kÅ/s. Then, a 30 nm layer of gold was deposited with the same background pressure and a deposition rate of ~2 kÅ/s. The substances for molecular wires, including the mercaptopyridine and all alkanethiol molecules, were purchased from Aldrich. The SAM patterns of the molecular wires were prepared via the microcontact printing method as reported previously^17^. In this process, a polydimethylsiloxane (PDMS) stamp was first dipped in a solution of the specific molecular wires (2 mM in ethanol) for 1 min and blown with N_2_ for 10 s. Then, the stamp was pressed down on a gold substrate for 3 s such that the molecules were transferred from the stamp to the substrate and formed the SAM patterns of the molecular wires. The SAM patterns of the multiple molecular wires, such as those in Fig. 3, could be prepared by repeating the stamping processes using different molecular wires.

### Mapping of the electrical currents and noise spectra

A conductive AFM (XE-70, Park Systems) in contact mode with a feedback loop was used with a conductive AFM probe based on Pt (25Pt300B from Park Systems, spring constant ~18 N/m) to map the electrical currents and noise spectra of the SAM patterns of the molecular wires. During the measurement process, the probe first made direct contact on the SAM with a contact force of ~1 μN, and then, a bias voltage was applied between the probe and underlying gold substrate using a function generator (DS345, Stanford Research Systems). During the measurements, the contact force between the AFM probe and sample was maintained at 1 μN using the contact force feedback loop based on a position-sensitive photo detector in the AFM system. The electrical currents through the probe were converted to amplified voltage signals by a low-noise preamplifier (SR570, Stanford Research Systems), and the noise PSD at a specific frequency range was measured by a homemade spectrum analyzer. The homemade spectrum analyzer consists of a band-pass filter and an RMS-DC converter. We used the band-pass filter included in the preamplifier SR570 to measure the signal within the desired frequency range. We built the RMS-DC converter circuit using an AD737 chip (Analog Devices). Finally, the noise PSD value at the central frequency of the measured frequency range was obtained by dividing the converted DC signals by the bandwidth of the used filter. The map of the electrical currents and noise PSDs was obtained by scanning the probe during the measurements with the AFM feedback on. The typical scanning speed was 0.5 μm/s. To achieve reliable mapping of PSD values at a specific frequency, the probe was held on each pixel of the sample for approximately 200 ms, and the data measured during the time period were averaged. For the measurement of the PSD spectra at different frequencies as shown in Fig. 2d, the probe was maintained at a specific point on the sample for 2 s, with feedback on for ~2 s, while measuring the currents through the probe, and an FFT network analyzer (SR770, Stanford Research Systems) was used to obtain the PSD spectra from the measured currents at different frequencies during the time period.

## Additional Information

**How to cite this article**: Cho, D. *et al*. Direct mapping of electrical noise sources in molecular wire-based devices. *Sci. Rep.* **7**, 43411; doi: 10.1038/srep43411 (2017).

**Publisher's note:** Springer Nature remains neutral with regard to jurisdictional claims in published maps and institutional affiliations.

## Supplementary Material

Supplementary Information
