# Supplementary material for: Direct mapping of electrical noise sources in molecular wire-based devices
Source: Sci Rep. 2017 Feb 24;7:43411. doi: 10.1038/srep43411 (PMC5324066; doi:10.1038/srep43411)
Supplement: Supplementary Information [file srep43411-s1.pdf]

[Supplementary Information]

Direct mapping of electrical noise sources in molecular wire-based devices

Duckhyung Cho[1], Hyungwoo Lee[1], Shashank Shekhar[1], Myungjae Yang[1], Jae Yeol Park[2], and Seunghun Hong[1,3]*

[1]Department of Physics and Astronomy, and Institute of Applied Physics, Seoul National University, Seoul 151-747, Korea

[2]Department of Automotive Engineering, Doowon Technical University College, Anseong 456-718, Korea

[3]Department of Biophysics and Chemical Biology, Seoul National University, Seoul 151-747, Korea

*Email: seunghun@snu.ac.kr

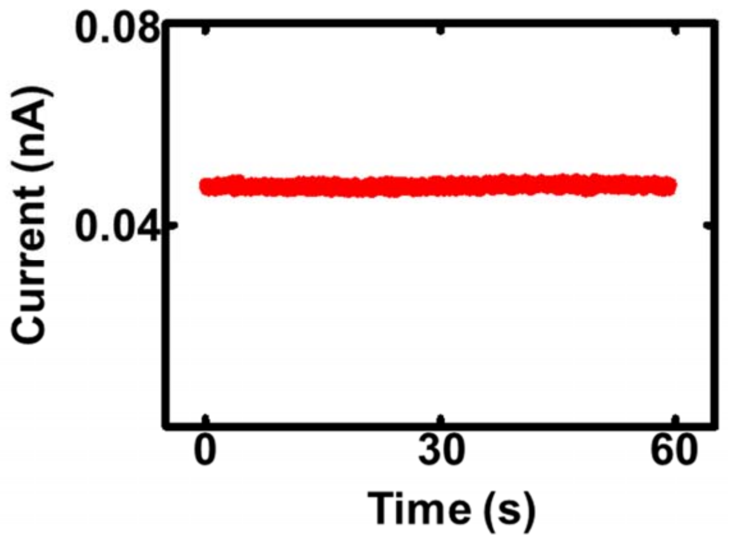

Figure S1. Long-term time-domain noise measurement. Time-domain current data measured over a one-minute period at a fixed position on a self-assembled monolayer (SAM) of C9 molecules. The data indicate a stable current level without any significant drift.

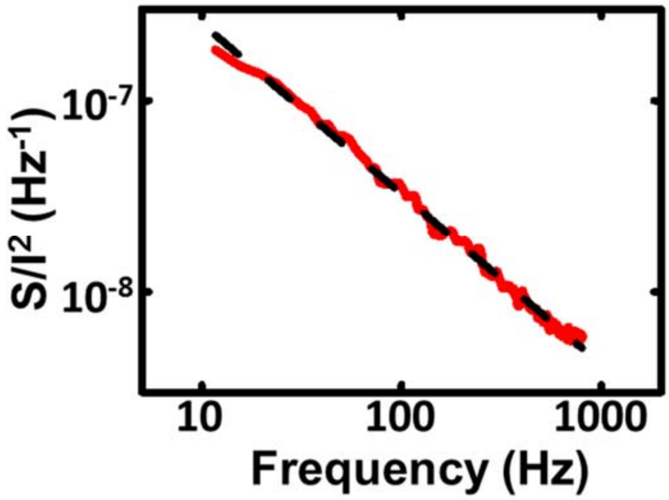

Figure S2. Noise spectrum in a resistor. Current-normalized PSD spectrum measured on a 1.0 GΩ glass glaze film resistor using our electronics system. The fitting curve, corresponding to a *1/f* spectrum, is indicated by a black dashed line.

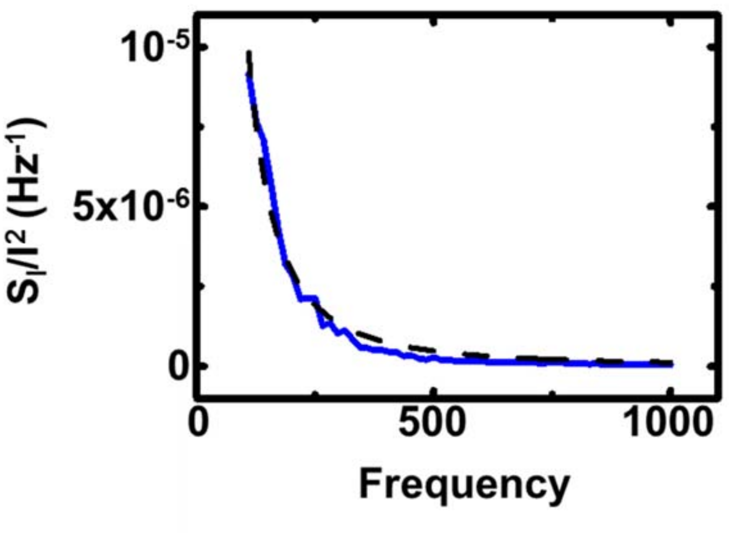

Figure S3. Frequency dependence of electrical noise in a molecular layer. Current-normalized PSD spectrum measured on a C9 SAM in Figure 2 (blue line) and the Lorentzian fitting of the spectrum (black dashed line). The fitting parameter τ was estimated as ~ 11 ms.

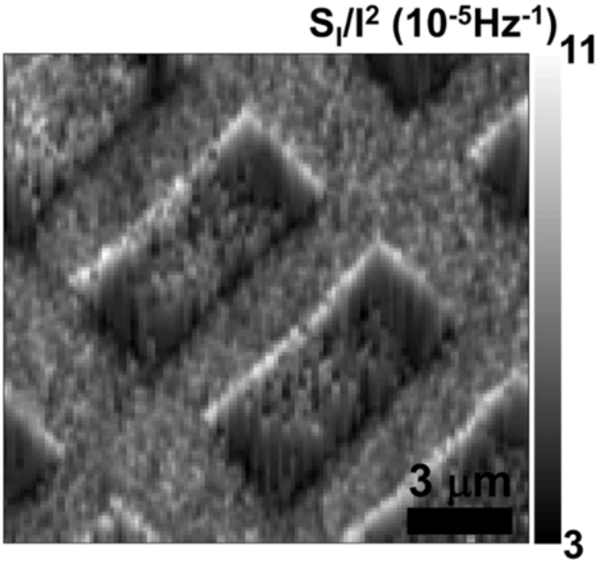

Figure S4. Noise mapping on a multi-molecule pattern. The current-normalized noise PSD (S_I/I^2) map (at 31.6 Hz) measured on the SAM patterns comprised of three different molecular wires. This map was obtained along with the current map of Figure 3b.

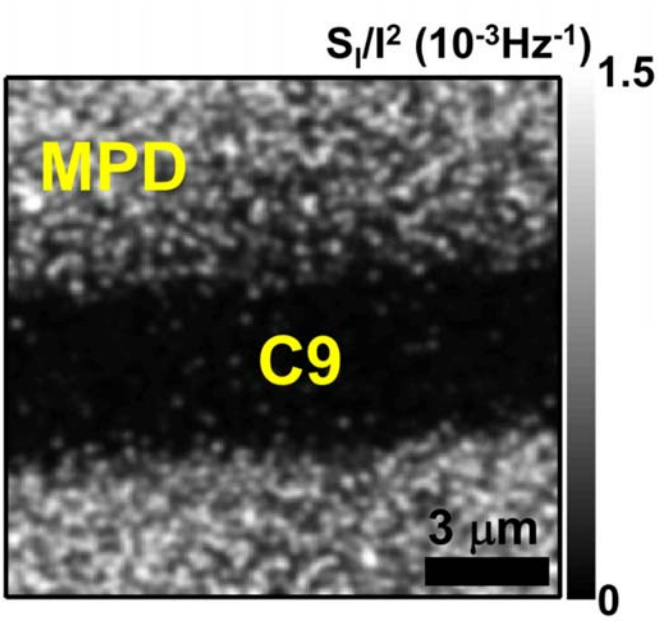

Figure S5. Noise mapping on a molecular pattern. S_I/I^2 map (at 31.6 Hz) measured on the MPD/C9 patterned sample in Figure 4.

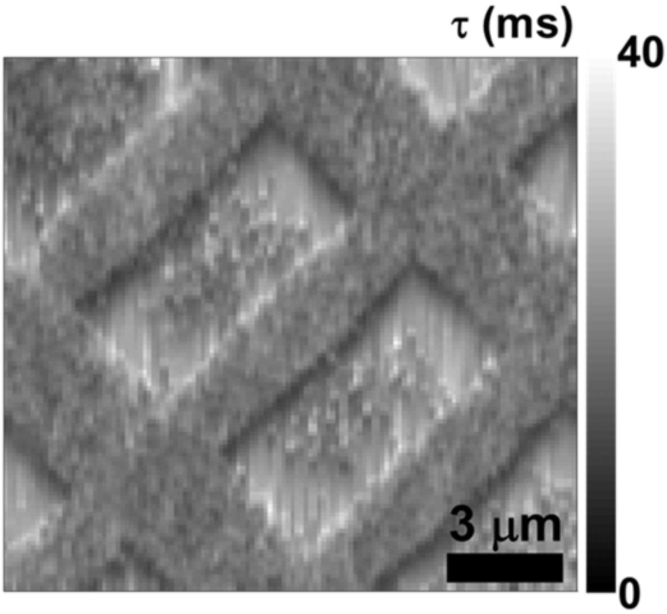

Figure S6. *τ* map on a molecular pattern. *τ* map calculated from the S_I/I^2 map (Figure S4) on the patterns of C8, C9, and C11 molecular wires. This map was obtained along with the current map of Figure 3b. The averaged τ values for the *C8*, *C9*, and *C11* molecular wires were *13*, *13*, and *24* ms, respectively.
